# Is it possible to estimate the minimal clinically important treatment effect needed to change practice in preterm birth prevention? Results of an obstetrician survey used to support the design of a trial

**DOI:** 10.1186/1471-2288-12-31

**Published:** 2012-03-19

**Authors:** Sue Ross, Jill Milne, Shannon Dwinnell, Selphee Tang, Stephen Wood

**Affiliations:** 1 Department of Obstetrics and Gynaecology, University of Calgary, Calgary T2N 2T9, Canada; 2 Department of Community Health Sciences, University of Calgary, Calgary T2N 4Z6, Canada; 3 Department of Family Medicine, University of Calgary, Calgary T2N 4N1, Canada; 4 Department of Surgery, University of Calgary, Calgary T2N 2T9, Canada

**Keywords:** Research design, Cross-sectional survey, Premature birth, Clinical trials as topic

## Abstract

**Background:**

Sample sizes for obstetrical trials are often based on the opinion of investigators about clinically important effect size. We surveyed Canadian obstetricians to investigate clinically important effect sizes required before introducing new treatments into practice to prevent preterm birth.

**Methods:**

Questionnaires were mailed to practicing obstetricians, asking the magnitude of pregnancy prolongation required to introduce treatments into practice. The three prophylactic treatments were of increasing invasiveness: vaginal progesterone, intramuscular progesterone, and cervical cerclage. We also asked about the perceived most relevant outcome measures for obstetrical trials and current obstetrical practice in preterm birth prevention.

**Results:**

544/1293(42.1%) completed questionnaires were received. The majority of respondents required one or two weeks' increase in length of gestation before introducing vaginal (372,77.1%), and intramuscular progesterone(354,67.9%). At least three weeks increase was required before introducing prophylactic cervical cerclage(326,62.8%). Clinicians who already used a treatment required a smaller difference before introducing it into practice. Decreasing neonatal morbidity was cited as the most important outcome for obstetrical trials (349,72.2%).

**Conclusion:**

Obstetricians would require a larger increase in treatment effect before introducing more invasive treatments into practice. Although infant morbidity was perceived as a more important outcome, clinicians appeared willing to change practice on the basis of prolongation of pregnancy, a surrogate outcome. We found that there is not a single minimum clinically important treatment effect that will influence all practising clinicians: rather the effect size that will influence physicians is affected by the nature of the treatment, the reported outcome measure and the clinician's own current clinical practice.

## Background

Clinical practice should be guided by evidence from well designed clinical trials [1-3]. Unfortunately, the transfer of knowledge from research into practice is often challenging, with a concomitant delay in the uptake of new evidence [3,4]. Many reasons for the delay have been proposed, including the inadequate dissemination of research findings [5,6], and barriers caused by entrenched physician beliefs [6-8].

Factors associated with research design also play an important role. Randomised controlled trials are generally accepted as the "gold standard" for health research, however studies that report statistically significant findings can lack relevance for major stakeholders such as clinicians, patients, and policy makers [9,10] and therefore fail to influence practice. Large sample sizes may lead to findings that are statistically significant but clinically irrelevant in their reflection of minor change [11-13]. Small sample sizes lead to studies that are underpowered to detect meaningful differences [12]. Additionally, sample size calculations may be based on estimates of effect size that are not relevant to the study being designed, or based on expert opinion [14]. Clinical relevance is important for fixed and other more efficient study designs (such as group-sequential designs [15]).

Concerns about clinical relevance of trials led to the concept of "minimal clinically important difference" (MCID) [16]. Originally applied to quality of life scales that are difficult for clinicians to interpret directly, MCID is defined as the lowest threshold of change believed to be important by patients and clinicians [13,16-19]. Other definitions have been suggested, including "minimal important difference" [14] and "really important difference" [20]. In treatment trials, important differences are termed "clinically important treatment effects" [21], however trial designers continue to struggle with determining the appropriate size of effect that would be sufficient to influence clinical practice. Many trialists choose to use an "opinion-based" method to estimate clinically important treatment effects, as opposed to "distribution approaches" which use statistical methods based on the distributions of scores of the measure of interest in control populations, or "anchor approaches" that compare scores of measures of interest with reference measures of known meaning [22]. The "opinion approach" gathers opinions of patients or experts [23], the investigators or their collaborators [24], however the effect size required to change practice is known to be affected by a number of additional factors including clinical context [17], physician background [25], and individual decision making patterns [26]. For this reason, reliance on expert opinion to determine clinically important effect size is unlikely to reflect the generality of clinicians who will ultimately be the recipients of the research findings.

In addition to other important factors such as the mean and variance of the primary outcome, a plausible estimate of effect size was a critical consideration in our estimation of sample size for a randomised trial of vaginal progesterone versus placebo to prevent preterm birth in multiple pregnancy [27]. We wished our trial to be as small as it could be to find the clinically important treatment effect, and to ensure our trial was feasible [26]. We knew that designing a large trial that resulted in a change in pregnancy prolongation of only one or two days (even if found statistically different) would be unlikely to change clinical practice, but we did not know the minimum difference that would influence obstetrical practice. We therefore undertook a survey of practicing obstetricians in Canada to examine the minimum prolongation of pregnancy necessary to change practice in hypothetical randomised controlled trials of treatments to prevent preterm birth. Our study also examined the relative importance of different outcome measures in clinical trials.

## Methods

This study, approved by the University of Calgary Conjoint Health Research Ethics Board (Ethics ID 18809), was a mailed questionnaire survey of all practicing obstetricians in Canada conducted between March 2006 and June 2006. Current lists of registered obstetricians and gynaecologists were obtained from the Colleges of Physicians and Surgeons in each Canadian province (n = 10) and territory (n = 3). The Ontario file excluded physicians who had not consented to have contact information released. The lists did not distinguish between obstetricians and gynaecologists, and therefore questionnaires were sent to all those listed (n = 1531).

Each identified physician was mailed a structured questionnaire. Two reminders were mailed at five week intervals to non-responders. Recipients were first asked to indicate if they did not provide care for pregnant women and return the questionnaire without further completion to avoid being sent further questionnaires.

The questionnaire was developed through discussion among the research team, and piloted with obstetrical residents (who were not therefore involved in the actual survey). The questionnaire consisted of four sections (Additional file 1). The first section contained three vignettes describing clinical settings in which treatments might be used for women at risk of preterm birth, and asked about the minimum prolongation of pregnancy that would be necessary for the respondent to introduce preventative treatments into their practice:

Treatment 1: prophylactic daily progesterone supplementation, administered vaginally after 16 weeks gestational age. Progesterone is a hormone treatment that may theoretically reduce the likelihood of preterm birth. Risk factor: multiple gestation.

Treatment 2: prophylactic weekly progesterone supplementation, administered intramuscularly after 16 weeks gestational age. Risk factors: history of preterm birth or shortened cervix on ultrasound or positive fetal fibronectin.

Treatment 3: prophylactic cervical cerclage placed between 16-23 6/7 weeks gestational age. Cerclage is a surgical procedure in which a stitch is placed in the cervix to prevent it from opening in pregnancy. Risk factor: shortened cervix on ultrasound.

The questionnaire provided three options for prolongation of pregnancy: one, two or three weeks. Respondents were asked to select the minimum prolongation they felt would be necessary to introduce that treatment into their clinical practice.

The next section asked about the most important clinical outcome for justifying a change to clinical practice, and whether the respondent would be willing to join randomised trials that would investigate the same three new prophylactic treatments. Respondents were also asked about their current practice in relation to the three treatments. Finally respondents were asked about their age, length of time in practice, gender and practice setting.

Statistical analyses were carried out using SAS 9.2 (SAS Institute Inc., Cary, NC), and were mainly descriptive. Analyses were carried out on only complete responses for each question, because there were several question funnels which meant that some questions were not relevant to all respondents. For example, in the sections about current clinical practice, it would have been inappropriate to impute data for subjects who did not respond because that section was not relevant to them. Friedman test, a non-parametric test comparing matched multiple groups, was used to test whether within subjects the responses (prolongation of pregnancy needed to change practice) differed significantly between the three interventions. Pearson Chi Squared tests were used to examine differences between groups (for example clinicians practicing in different settings) in the proportion of respondents giving different choices of gestational prolongation required to change practice.

## Results

### Response rate

Of 1531 questionnaires mailed out, 269 respondents did not provide care to pregnant women or the questionnaires were returned because the addressees had moved away. Thus questionnaires were presumed delivered to 1292 appropriate recipients, of whom 544 (42.1%) returned completed questionnaires. The characteristics of respondents are shown in Table 1.

**Table 1:** Characteristics of respondents

Characteristic	Unit/details	Description (n = 544)	# missing
**Age**	mean years	46 (SD 10)	9

**Time in practice**	mean years	15 (SD 10)	4

**Gender**	male female	272 (50.6%) 266 (49.4%)	6

**Practice setting**	urban/suburban small town/rural	428 (80.0%) 107 (20.0%)	9

**Hospital**	teaching community other	283 (52.2%) 232 (42.8%) 27 (5.0%)	2

### Prolongation of pregnancy necessary to change practice

Respondents were asked to select the minimum prolongation of pregnancy they believed was necessary to introduce a preventative treatment into their clinical practice for patients with risk factors for preterm birth (Table 2). The majority of respondents would accept a minimum increase in gestation of one week or two weeks to introduce vaginal progesterone for women with multiple gestations (372, 77.1%), and intramuscular progesterone for women with singleton pregnancies and a history of preterm birth or shortened cervix on ultrasound or positive fetal fibronectin (354, 67.9%). Prolongation of pregnancy by at least 3 weeks was required by the majority of respondents (326, 62.8%) before introducing prophylactic cervical cerclage for women with a short cervix.

**Table 2:** Prolongation of pregnancy required to introduce new treatment into practice

	Treatment used in clinical practice		
Prolongation of pregnancy required to change practice	Progesterone PV in multiples (n = 544)	Progesterone IM in singletons (n = 544)	Cerclage (n = 544)
**1 week**	165 (31.5%)	138 (26.5%)	62 (11.9%)

**2 weeks**	207 (39.6%)	216 (41.5%)	131 (25.2%)

**3 weeks**	151 (28.9%)	167 (32.1%)	326 (62.8%)

**Missing**	21	23	25

A Friedman test was carried out to compare, within subjects, the responses for the three interventions. This test found that responses for the three interventions differed significantly (×^2 ^= 291, df = 2, *p *< 0.001). The mean ranks for the three treatments were: vaginal progesterone 1.75, intramuscular progesterone 1.85, cerclage 2.39. This suggests that there was a tendency to need a larger effect size to introduce intramuscular progesterone than vaginal progesterone, and there was a need for a larger effect size to introduce cerclage.

Pearson Chi Squared tests were carried out to estimate if there was a difference between respondents from teaching hospitals versus those in community hospitals in the level of evidence required to change their practice for each of the three treatments. These tests did not demonstrate differences between groups.

### Current practice

Respondents were asked to describe their current obstetrical practice relevant to the treatments in the scenarios (Table 3).

**Table 3:** Current clinical practice - treatments for women at risk of preterm birth

		Treatment used in clinical practice		
**Current clinical practice**		**Progesterone supplementation** **in multiple pregnancies** **(n = 544)**	**Progesterone supplementation** **in singleton pregnancies** **(n = 544)**	**Cerclage** **(n = 544)**

use this treatment		17 (3.2%)	51 (9.7%)	317 (62.8%)

do not use this treatment		520 (96.8%)	476 (90.3%)	188 (37.2%)

missing		7	17	39

**For 'YES' ONLY**		**n = 17**	**n = 51**	**n = 317**

Risk factors influencing decision *				

previous preterm delivery		17 (100.0%)	49 (96.1%)	176 (55.5%)

hort cervix		12 (70.6%)	20 (39.6%)	190 (59.9%)

previous surgery		6 (35.3%)	8 (15.7%)	96 (30.3%)

previous PPROM		9 (52.9%)	24 (47.1%)	38 (12.0%)

positive FFN test		8 (47.1%)	12 (23.5%)	8 (2.5%)

multiple gestation		*not noted*	*not noted*	20 (6.3%)

incompetent cervix		*not noted*	*not noted*	139 (43.8%)

Route of administration favoured *	oral	4 (23.5%)	14 (27.5%)	*not applicable*

	IM	3 (17.6%)	7 (13.7%)	

	vaginal	11 (64.7%)	37 (72.5%)	

**Earliest gestational age treatment** **starts**	mean weeks	12 (SD 8) range 2 to 30 missing 1	13 (SD 5) range 4 to 24 missing 1	*surgery not before:* 13 (SD 2) range 7 to 18 missing 23

**Latest gestational** **age treatment stops**	mean weeks	27 (SD 10) range 12 to 36 missing 1	29 (SD 8) range 12 to 36 missing 1	*surgery not after: *22 (SD 4) range 12 to 34 missing 47

Note: * respondents could check more than one risk factor and treatment type

Only 17 (3.2%) currently prescribed prophylactic progesterone for women with multiple pregnancies, all of whom would consider a history of previous preterm delivery in their decision to treat. Among the 17 who would use progesterone, vaginal treatment was prescribed by 11 (64.7%), with the earliest start at a mean of 12 weeks' gestation and the latest stopping date at mean 27 weeks.

Fifty-one respondents (9.7%) would prescribe progesterone for women with risk factors with singleton pregnancies, the majority of whom (49, 96.1%) would consider a history of previous preterm delivery in their decision to treat. Similarly, vaginal treatment was favored by the majority (37 (72.5%), with the earliest start at a mean of 13 weeks gestation and the latest stopping at mean of 29 weeks.

Cerclage was a more widely used treatment, with 317 (62.8%) responding that they currently perform prophylactic cervical cerclage. A number of risk factors influenced the decision to place a cerclage: the most common were short cervix (190, 59.9%), previous preterm delivery (176, 55.5%) and a diagnosis of incompetent cervix (139, 43.8%). Cerclage would be performed at the earliest at mean 13 weeks of gestation, and at the latest at 22 weeks.

Pearson Chi Squared tests were carried out to estimate if there was a difference between respondents who currently used a treatment versus those who did not, in the level of evidence required to change their practice. The test for cerclage appeared to indicate that more who currently used prophylactic cerclage would be willing to accept a smaller effect size to introduce prophylactic cerclage into practice (Table 4, ×^2 ^= 8.17, *p *= 0.017). For progesterone treatments, current use was not associated with effect size.

**Table 4:** Current use of cerclage and minimum prolongation of pregnancy required to introduce prophylactic cerclage into practice

	Current use of cerclage	
**Prolongation of pregnancy** **required to change practice**	**Use cerclage** **(n = 308)**	**Do not use cerclage** **(n = 174)**

**1 week**	44 (14.3%)	15 (8.6%)

**2 weeks**	86 (27.9%)	36 (20.7%)

**3 weeks**	178 (57.8%)	123 (70.7%)

### Feasibility of a trial

The majority of respondents stated that decreased fetal morbidity (379, 72.2%) was the single most important outcome measure that would justify introducing a new treatment into their practice, rather than prolongation of pregnancy (95, 18.1%) (Table 5).

**Table 5:** Feasibility of trials

What would be the best outcome measure for a trial? (n = 544)			
	**Outcome**	**Response**	
	prolongation of gestation		95 (18.1%)
	decreased fetal morbidity		379 (72.2%)
	decreased fetal mortality		50 (9.5%)
	decreased maternal morbidity		1 (0.2%)
	missing		19
**Would you take part in a trial?**			
	**Vaginal Progesterone** **in multiple** **pregnancies** **(n = 544)**	**Progesterone IM in** **singleton pregnancies** **(n = 544)**	**Cerclage** **(n = 544)**

yes	450 (84.1%)	410 (77.5%)	239 (45.5%)
no	85 (15.9%)	119 (22.5%)	288 (54.6%)
missing	9	15	17

The majority of respondents would be willing to take part in a placebo controlled trial of vaginal progesterone for women with multiple pregnancies (450, 84.1%), and a trial of intramuscular progesterone for women with singleton pregnancies (410, 77.5%) (Table 6). The most commonly cited reason for not joining the multiple gestation trial was having too few such patients in their practice (26/85, (30.6%) (Table 6). The main concern about the intramuscular progesterone trial was patient discomfort associated with weekly intramuscular injections (32/119, 26.9%) (Table 6). By contrast, only a minority of respondents would consider taking part in the proposed cerclage trial (239, 45.5%) (Table 5): the main reason for not wishing to join such a trial was concerns about the invasive nature of the cerclage itself (110/288, 38.2%) (Table 6).

**Table 6:** Reasons given for not taking part in a trial

Trial	Reasons for not joining	
**Vaginal progesterone in** **women with multiple** **pregnancies**		n = 85
	Small numbers/rural	26 (30.6%)
	Concerns re intervention	11 (12.9%)
	Concerns re trial design	10 (11.8%)
	Retiring soon	9 (10.6%)
	Lack of resources	9 (10.6%)
	Do not do research	6 (7.1%)
	Not recorded	14 (16.5%)

**IM progesterone in women** **with singleton pregnancies with** **risk factors for preterm birth**		n = 119
	Concerns re intervention	32 (26.9%)
	Lack of resources	23 (19.3%)
	Small numbers/rural	18 (15.1%)
	Concerns re trial design	9 (7.6%)
	Retiring soon	9 (7.6%)
	Do not do research	4 (3.4%)
	Sufficient evidence	2 (1.7%)
	Not recorded	22 (18.5%)

**Cerclage in women with risk** **factors for preterm birth**		n = 288
	Concerns re intervention	110 (38.2%)
	Sufficient evidence	38 (13.2%)
	Do not do cerclage	32 (11.1%)
	Concerns re trial design	28 (9.7%)
	Small numbers/rural	21 (7.3%)
	Lack of resources	10 (3.5%)
	Retiring soon	8 (2.8%)
	Do not do research	4 (1.4%)
	Not recorded	37 (12.8%)

## Discussion

The determination of a suitable estimate of effect size is notoriously difficult [26]. Our mailed survey of all Canadian obstetricians attempted to estimate the clinically important treatment effect required to change practice in the prevention of preterm birth, in order to increase the likelihood that our research would be relevant, with the potential to influence clinical practice. Our study found that responding clinicians were willing to identify an increase in duration of pregnancy that they believed would change their practice. The small amount of missing data indicated that respondents found the clinical scenarios plausible.

Our study found that minimal clinically important treatment effect was associated with the invasiveness of the treatment, and current clinical practice of the respondents. A one or two week prolongation of pregnancy was the minimum effect size required by around 70% of obstetricians to introduce new progesterone treatments into their practice for treating women at risk of preterm birth. Cerclage, a more invasive treatment, required a larger minimum effect size: a three week increase was needed to introduce this treatment. Availability of the treatment also influenced the minimum important treatment effect: clinicians who already had access to cerclage were willing to accept a smaller effect size to utilize this treatment.

The impact of the choice of effect size may best be illustrated using the parameters we employed when calculating the sample size for our progesterone trial [27]. For that study, we used local data from a two year period to estimate the usual (untreated) mean and standard deviation gestational age of delivery for multiple pregnancies. Sample size calculations using a power of 80% and two-sided significance level of 0.05, would estimate sample sizes as follows: to detect a three week difference, a sample of 36 (18 per randomised group) would be required; for two weeks, the sample size would be 78 (39 per group); for one week, the sample size would be 156 (78 per group). If the three week effect size was chosen, and therefore the smaller sample size was used, a real difference between treated and control groups of one or two weeks would not be identified as a significant difference. These examples clearly show that the choice of clinically important treatment effect has a practical effect on the design, conduct and clinical relevance of the study. Applying these examples to our hypothetical scenarios, it is clear that a trial of cerclage versus no cerclage would be smaller in size than a trial of a progesterone treatment versus placebo.

The principal goal of prophylactic treatment for women at risk of preterm labour is to improve the outcome for the neonate by reducing morbidity and mortality. The consequences of even late preterm birth are considerable, with worse developmental outcomes and academic difficulties up to seven years of age compared to term infants [28]. In our questionnaire study, the majority of respondents (72.2%) clearly recognised this by stating that decreased fetal morbidity was the most important outcome to justify changing clinical practice. Despite this, in our hypothetical scenarios where clinicians were asked to consider changing their clinical practice on the basis of prolongation of pregnancy, all but 4% were willing to make a choice based on that outcome. It therefore appears that clinicians would be willing to accept a surrogate outcome on which to base a change in clinical practice. This is an important point: a trial that measures impact of a treatment on fetal or neonatal morbidity, even in a trial examining preterm birth where adverse neonatal events would be expected, will need to be far larger than one that measures impact on pregnancy duration, where every pregnancy will have an outcome [29]. In addition, the definition of a 'neonatal morbidity' composite outcome is fraught with difficulty, and is open to misinterpretation by clinicians and patients [29]. Thus the choice of effect size and choice of outcome will impact on the size and expense of a clinical trial. Both of these choices will also impact on the adoption of trial findings into clinical practice.

We were interested about the feasibility of trials of treatments to prevent preterm birth. Concern for the patient and increasing invasiveness of the intervention impacted on respondents' decisions about taking part in a trial. The majority of respondents would be willing to take part in both hypothetical progesterone trials. The most frequently cited reason for declining to take part in the multiple trial was feasibility (lack of suitable cases), while concern about the intervention (for example patient discomfort) was most commonly cited for the intramuscular progesterone trial. Concern about cerclage itself was a common reason for not wishing to take part in a trial, although 13.2% believed that there was already sufficient evidence to use prophylactic cerclage in women with a short cervix. We did not lead clinicians in their reason for not wishing to take part in each trial, rather leaving them to provide text answers to open-ended questions. Therefore we believe the responses were those that mattered most to the clinicians.

The design of our questionnaire could be criticised for a number of reasons. Firstly, the questionnaire described three hypothetical scenarios, including different risk factors for preterm birth. Although designed to represent clinical experience, we do not know whether the responses would reflect actual practice. Secondly, in the clinical scenarios, we used a forced choice method that asked clinicians to choose a one, two or three week prolongation of pregnancy as the outcome for the hypothetical treatments. It is possible that clinicians would be willing to accept a shorter increase or would prefer a longer increase in gestation before changing their practice. We set the earliest gestational age for treatment at 16 weeks, and the longest duration of pregnancy as 33 weeks and 4 days [30,31], for each hypothetical prolongation of pregnancy. Thus it is possible that the choice of clinically important increase in duration of pregnancy may have been affected by our descriptions. These scenarios and choices were necessarily complex, reflecting situations commonly encountered by obstetricians who manage high risk pregnancies.

Much research relevant to the prevention of preterm birth has been published since our survey was carried out, reflecting the ongoing interest and importance of this topic. Recent publications conclude that, in appropriately selected women with high-risk singleton pregnancies, progesterone can reduce preterm birth before 33 weeks' gestation [32], and cerclage can reduce preterm birth and improve neonatal outcome [33]. Trials of progesterone in multiple pregnancies have failed to prevent preterm birth [34], most likely because of a difference in aetiology, perhaps involving larger fetal and placental mass, and greater stretching of uterine muscle [35]. Nonetheless, preterm birth remains the most concerning perinatal problem for obstetricians, and therefore new progesterone trials continue to be undertaken [36], and the search for new effective treatments continues.

Our survey of all obstetricians was challenging and would not be feasible for all trials, partly because such surveys are costly and time consuming, and partly because clinicians would be plagued by many similar questionnaires, leading to declining response rates and possible bias in the results. In the future, we believe that a more limited survey of a random sample of practicing clinicians would be more appropriate. In our study, however, obstetricians were willing to respond to our fairly complex questionnaire: we achieved a response rate of 42.1% after only two reminders, similar to response rates from other surveys of clinical practice [25,37,38]. Our respondents came from a range of clinical settings and had a range of experience. Our large number of responses (n = 544) leads us to believe that our findings are more representative of clinician beliefs than the more usual practice of identifying the minimum clinically important treatment effect by discussion with trial collaborators [25] or experts [23].

## Conclusion

The importance of our study lies in the continuing need to undertake meaningful and efficient randomized trials that are designed to support clinical practice [2,3]. Such trials must provide clinically important differences rather than merely statistically significant differences, and the results should be available in a timely way [7,13]. To be timely, trials should be designed to be as small as realistically possible to obtain clinically relevant results. Our study developed a method that was able to estimate the clinically important difference needed to design our randomised trial [27]. Clinical trialists must make the additional effort to estimate the minimum clinically important treatment effect that is relevant to practicing clinicians. Without this effort, trials will continue to fail to influence clinical practice.

## Competing interests

The authors declare that they have no competing interests.

## Authors' contributions

All authors contributed equally to the design of the follow-up study, participated in writing the manuscript and have read and approved the final version.

## Authors' information

SR is a health services researcher and Professor in the Departments of Obstetrics and Gynaecology, Family Medicine, Community Health Sciences and Family Medicine, and Director of Research in the Department of Obstetrics and Gynaecology, University of Calgary. JM was a research associate in the Department of Obstetrics and Gynaecology, University of Calgary. SD was involved in this study during her residency in obstetrics and gynaecology in Calgary, and is now a perinatologist in the Department of Obstetrics and Gynaecology at the University of Calgary. ST is data analyst in the Department of Obstetrics and Gynaecology, University of Calgary. SW is an obstetrician/gynaecologist and Associate Professor in the Departments of Obstetrics and Gynaecology and Community Health Sciences, University of Calgary.

## Pre-publication history

The pre-publication history for this paper can be accessed here:

http://www.biomedcentral.com/1471-2288/12/31/prepub

## Supplementary Material

Additional file 1 **Questionnaire**. Click here for file
