# Supplementary material for: Is it possible to estimate the minimal clinically important treatment effect needed to change practice in preterm birth prevention? Results of an obstetrician survey used to support the design of a trial
Source: BMC Med Res Methodol. 2012 Mar 19;12:31. doi: 10.1186/1471-2288-12-31 (PMC3364141; doi:10.1186/1471-2288-12-31)
Supplement: Additional file 1 — Questionnaire. [file 1471-2288-12-31-S1.PDF]

Additional material

Questionnaire

UNIVERSITY OF CALGARY

The Prolongation of Pregnancy Necessary to Change Antenatal Management of Patients at High Risk for Preterm Delivery: a survey of Canadian obstetricians.

If you do not provide care for pregnant women, PLEASE mark this box and return the survey so that we do not trouble you again<br>□ Do not provide care for pregnant women

Section I:

Q.1 ***At minimum***, which magnitude of prolongation of gestation would be necessary for you to introduce the three following preventative treatments into your clinical practice for patients with risk factors for preterm birth?
---please check ***one*** box per treatment option

Treatment 1: prophylactic *daily* progesterone supplementation, administered *vaginally* after 16 weeks gestational age

Risk factor: multiple gestation

please check one option

□ 1 week prolongation of gestation (eg, 32 4/7→ 33 4/7)	□ 2 week prolongation of gestation (eg, 31 4/7→ 33 4/7)	□ 3 week prolongation of gestation (eg, 30 4/7→ 33 4/7)

Treatment 2: prophylactic *weekly* progesterone supplementation, administered *intramuscularly* after 16 weeks gestational age

Risk factors: history of preterm birth *or* shortened cervix on ultrasound *or* positive fetal fibronectin

please check one option

□ 1 week prolongation of gestation (eg, 32 4/7→ 33 4/7)	□ 2 week prolongation of gestation (eg, 31 4/7→ 33 4/7)	□ 3 week prolongation of gestation (eg, 30 4/7→ 33 4/7)

Treatment 3: prophylactic cervical cerclage placed between 16-23 6/7 weeks gestational age

Risk factor: shortened cervix on ultrasound

please check one option

□ 1 week prolongation of gestation (eg, 32 4/7→ 33 4/7)	□ 2 week prolongation of gestation (eg, 31 4/7→ 33 4/7)	□ 3 week prolongation of gestation (eg, 30 4/7→ 33 4/7)

Q.2 If a well-designed randomized controlled trial demonstrated that a treatment was superior to placebo for the prevention of preterm birth, an improvement in *which of the following outcome measures* would you consider to be ***most important*** for you to justify introducing this treatment into your clinical practice?
---please check only ***one*** box

☐ Prolongation of gestation
☐ Decreased fetal morbidity
☐ Decreased fetal mortality
☐ Decreased maternal morbidity

Section II: Feasibility

Q.1 Would you participate in a randomized controlled trial administering *daily vaginal progesterone* (vs. placebo) to patients pregnant with multiples to determine whether it will prolong gestation?

YES ☐ NO ☐

---If NO, why not?

____
____

Q.2 Would you participate in a randomized controlled trial administering *weekly intramuscular progesterone* (vs. placebo) to patients with risk factors for preterm delivery to determine whether it will prolong gestation?

YES ☐ NO ☐

---If NO, why not?

____
____

Q.3 Would you participate in a randomized controlled trial performing *cervical cerclage* (vs. no cerclage) on patients with short cervix on ultrasound to determine whether it will prolong gestation?

YES ☐ NO ☐

---If NO, why not?

____
____

Section III: Current clinical practice
Q.1 Is it your current clinical practice to use prophylactic progesterone supplementation in patients pregnant with *multiples*?

YES □ NO □

----If yes, what risk factors influence your decision to start prophylactic progesterone supplementation in patients pregnant with *multiples*?

Previous preterm delivery	YES □	NO □
Short cervix on US	YES □	NO □
Previous cervical surgery	YES □	NO □
Previous PPROM	YES □	NO □
Positive fetal fibronectin	YES □	NO □

Other risk factors? Please specify ___________________________________

----If yes, what *route of administration* do you prescribe?

Oral □ Intramuscular □ Vaginal □

----If yes, what is the *earliest* gestational age that you start treatment and what is the *latest* gestational age that you stop treatment?

Start treatment _____ weeks GA (*earliest GA)*
Continue treatment *until* _____ weeks GA *(latest GA)*

Q.2 Is it your current clinical practice to use prophylactic progesterone supplementation in patients pregnant with *singletons* who have risk factors for preterm birth?

YES □ NO □

----If yes, what risk factors influence your decision to start prophylactic progesterone supplementation in patients pregnant with *singletons*?

Previous preterm delivery	YES □	NO □
Short cervix on US	YES □	NO □
Previous cervical surgery	YES □	NO □
Previous PPROM	YES □	NO □
Positive fetal fibronectin	YES □	NO □

Other risk factors? Please specify ___________________________________

----If yes, what *route of administration* do you prescribe?

Oral □ Intramuscular □ Vaginal □

----If yes, what is the earliest gestational age that you start treatment and what is the latest gestational age that you stop treatment?

Start treatment _____ weeks GA (*earliest GA)*

Continue treatment *until* _____ weeks GA *(latest GA)*

Q.3 Is it your current clinical practice to perform prophylactic cervical cerclage?

YES ☐ NO ☐

----If yes, what risk factors influence your decision to perform cervical cerclage in pregnant patients?

Previous preterm delivery	YES ☐	NO ☐
Short cervix on US	YES ☐	NO ☐
Previous cervical surgery	YES ☐	NO ☐
Previous PPROM	YES ☐	NO ☐
Positive fetal fibronectin	YES ☐	NO ☐
Multiple gestation	YES ☐	NO ☐

Other risk factors? Please specify ___________________________________

----If yes, *at what gestational age* do you perform cerclage?

Not before _____weeks GA

Not after _____ weeks GA

Section IV: General Information

Q.1 What is your present age? ___ years

Q.2 How many years have you been providing obstetrical care? ___ years

Q,3 What is your gender? ☐ male ☐ female

Q.4 How would you describe your practice setting? ☐ urban/suburban ☐ small town/rural

Q.5 Is your hospital a: ☐ teaching hospital ☐ community hospital ☐ other

(*if `other' please specify*) __________________________

Thanks for completing the questionnaire!

Please return it in the enclosed stamped addressed envelope to:

Shannon Dwinnell, Foothills Medical Centre (room 404), 1441 – 29th Street NW, Calgary, AB T2N 4J8
